# Medication Adherence Among Multimorbid Patients With Polypharmacy and Its Relation to Social Support at National Guard Primary Health Care Centers, Riyadh

**DOI:** 10.7759/cureus.30679

**Published:** 2022-10-25

**Authors:** Ahmed S Almutairi, Taghreed M Alhazmi, Yazeed H Alotaibi, Abdulmajeed A Alfraidi, Abdulaziz M Alsaad, Rashed A Matrood, Abdulmohsen N Al-khatir, Abdulrahman A Alsubaie, Waleed M Alotibi

**Affiliations:** 1 College of Medicine, King Saud Bin Abdulaziz University for Health Sciences, Riyadh, SAU; 2 Family Medicine and Primary Care, King Abdulaziz Medical City, Riyadh, SAU

**Keywords:** multimorbidity, social support, medication, polypharmacy, adherence

## Abstract

Objective: The aim of the present study was to estimate the prevalence of nonadherence to medication in multimorbid patients with polypharmacy and its relationship to social support in primary healthcare centers in Riyadh, Saudi Arabia.

Methods: We conducted a cross-sectional, convenience-sample, non-randomized study in three primary healthcare centers managed by National Guard Health Affairs. The participants included 417 adult patients - (a) with two or more chronic illnesses and (b) who were taking two or more medications. The primary outcome variable was the prevalence of medication nonadherence in multimorbid patients with polypharmacy as measured by the modified Morisky Medication Adherence Scale (MMAS-8). The second main variable was the impact of functional social support, as measured by the Duke-UNC Functional Social Support Questionnaire (FSSQ), on medication adherence.

Results: The level of medication adherence was low for 194 (46.5%) of the 417 patients, medium for 127 (30.5%), and high for 96 (23%). There were 256 (61.4%) male participants and 161 (38.6%) females, and their mean age was 59.15 (SD ± 11.186) years. Additionally, 171 (41%) participants used two or three medications, 127 (30.5%) used four or five medications, and 119 (28.5%) used more than five medications; 178 (42.7%) of the patients had two comorbidities, 136 (32.9%) had three comorbidities, 69 (16.5%) had four comorbidities, and 31 (7.5%) had five comorbidities.

Some social support data from the Duke-UNC Functional Social Support Questionnaire (FSSQ) was missing for 58 (13.9%) of the participants. Among the rest of the sample, reported levels of social support levels were high for 246 (59%) patients, medium for 101 (24.2%), and low for 12 (2.9%) patients. None of the differences between social support and medication adherence were statistically significant. However, 61 (24.8%) patients reported both high social support and high medication adherence; 173 (48.2%) had low social support and low medication adherence (p = 0.470).

Conclusion: We found that medication nonadherence in multimorbid patients with polypharmacy was high (46.5%). Although there were no statistically significant relationships between social support and medication adherence, certain patient characteristics were associated with low medication adherence - age over 60 years, male gender, and number of medications.

## Introduction

Noncommunicable diseases, which primarily include cardiovascular diseases, cancer, respiratory diseases, and diabetes, are a growing global problem affecting all age groups [1]. As population age, the prevalence of chronic diseases rise. According to the World Health Organization (WHO), multimorbidity is defined as “people who have two or more chronic health conditions that are often long term that require complex and ongoing care” [2]. A major study in a developed country found that more than 40% of the population (all ages) had at least one long-term health condition, with approximately 25% having multiple long-term health conditions [2]. Furthermore, a study in Cyprus estimated that among adults of age 65 years or older, the average number of patients with multiple chronic diseases was approximately 68.9% [3]. A study published in Buraydah in Al-Qassim, Saudi Arabia, demonstrated that 34.5% of the participants had two or more chronic diseases [4]. Another study in Saudi Arabia indicated a significant increase in the incidence of non-communicable diseases over the last decade [5]. For example, diabetes in men increased from 9.7% in 1990 to 34.7% in 2017, demonstrating that multimorbidity is a common health issue in Saudi Arabia and worldwide.

To control illness and improve quality of life, a patient with comorbidities is often prescribed multiple and complex drug regimens (polypharmacy). The definition of polypharmacy varies. According to a systemic review of polypharmacy definitions, the use of multiple medicines, commonly referred to as polypharmacy, is common in the older population with multimorbidity, as one or more medicines may be used to treat each condition [6]. One of the most common definitions is the simultaneous use of five or more medications daily - a condition that can result in unfortunate events, especially among older adults [6]. Adverse drug reactions (ADRs) caused by decreased drug clearance and metabolic changes associated with aging are among the most concerning events [7]. Additionally, polypharmacy has been shown to reduce medication adherence and quality of life; thus, identifying and avoiding polypharmacy might contribute to better outcomes and greater quality of life for older adults [7]. According to one study in Qatar, the prevalence of polypharmacy in the elderly was 75.5% [8].

Nonadherence to prescribed medications is becoming increasingly common among patients of all ages, particularly the elderly. Nonadherence refers to any practice that does not follow medical advice or the incorrect use of medication in terms of time, dose, and frequency; it has been linked to a variety of factors, including polypharmacy and a lack of social support [9]. Furthermore, the individual’s living situation, level of education, income, and social support all have an impact on patient compliance [10]. The global rate of poor adherence is 46.6% [11]. A study on adherence to hypertension medication in Alsaqabi, Saudi Arabia, found that 38.8% of patients did not adhere to the prescribed medication regimen [12]. Another factor that may contribute to nonadherence is a lack of social support, having someone to talk to or ask for help, such as a family member, friend, or neighbor [13]. The presence of social support has a variety of effects on one’s health, particularly in patients with comorbidities. For example, one study found that social support can play a positive role in the treatment of some diseases, such as hypertension, by lowering the risk of high blood pressure [14]. Another positive effect of social support is better adherence to medication and medical advice [15]. The aim of the present study was to estimate the prevalence of nonadherence among patients with multimorbidity with polypharmacy and its relationship to social support in primary healthcare centers.

## Materials and methods

This cross-sectional, convenience sample, non-randomized study was conducted in three primary healthcare centers managed by the National Guard Health Affairs - Health Care Specialty Center, King Abdulaziz City Housing, and National Guard Comprehensive Specialized Clinic. Each center has a variety of clinics, including family medicine, obstetrics and gynecology, and pediatrics. The research was conducted in family medicine clinics. Any adult patient with two or more chronic diseases and taking two or more medications was eligible for the study. Patients with mental illnesses or those who worked as healthcare personnel were excluded from the study. As there was no data on the percentage of multimorbidity in Saudi Arabia or the Middle East, the study sample size was calculated using the data from a study conducted in the United States. The US population with multimorbidity was 55,411 representing 0.021% of the adult population in the United States [16] and 0.021% of the Saudi adult population (26,456,921) is approximately 5,555 people [17].

The frequency of nonadherence to polypharmacy was required to estimate the sample size, which was found to be approximately 40.8% in another study [15]. We used OpenEpi (Seattle, WA: The Bill & Melinda Gates Foundation) to calculate the required sample size of 348, with a confidence level of 95%. The sample size was adjusted to 420 to account for the missing data. Data were collected through face-to-face interviews. The data collectors were present in the clinic and invited any patient who met the inclusion and did not meet the exclusion criteria to take part in the study. Questionnaires were translated into Arabic. The experts performed back translation to determine the accuracy of the instruments.

The main outcome variable was the prevalence of medication nonadherence in multimorbid patients with polypharmacy, using a modified Morisky Medication Adherence Scale (MMAS-8) [18]. The items included questions, such as “Do you sometimes forget to take your pills?” “Thinking back over the past two weeks, were there any days when you did not take your medicine?” “Have you ever cut back or stopped taking your medication without telling your doctor because you felt worse when you took it?” “When you travel or leave home, do you sometimes forget to bring along your medication?” “Did you take your medicine yesterday?” “When you feel like your condition is under control, do you sometimes stop taking the medication?” “Do you ever feel hassled about sticking to your treatment plan?” and “Do you have difficulty in remembering to take all of your medications?” Response choices for items 1 to 7 were “yes” or “no”; item 8 was answered using a five-point Likert response choice. Most ‘‘no” responses were rated ‘‘1” and ‘‘yes” responses were rated ‘‘0.” Exceptions include item 5, for which a ‘‘yes” response was rated as ‘‘1” and ‘‘no” was rated as ‘‘0,” and item 8, wherein the response ‘‘0” (never) was scored as ‘‘1,” the response ‘‘4” (always) was scored as ‘‘0,” and responses ‘‘1,” “2,” and “3” were rated as ‘‘0.25,” “0.75,” and “0.75,” respectively (Appendix 1). Total MMAS-8 scores ranged from 0 to 8 and were divided into three levels of adherence - high adherence (score = 8), medium adherence (score from 6 to <8), and low adherence (score <6) [18]. We also included other grouping variables, such as age, sex, education level, marital status, and household income, to examine their relationship with medication adherence.

The second major variable was the impact of structural social support on medication adherence, as measured by the Functional Social Support Questionnaire (FSSQ) [19]. The items include statements, such as “I have opportunities to talk to someone I trust about my personal life and family; I have opportunities to talk to someone about money problems; I have opportunities to talk to someone about problems at work or home; I receive love and affection; I have people who care what happens to me; I receive help when I am sick in bed; I receive useful advice about important things in my life; I receive help in matters related to my home; I am visited by friends and family; I receive invitations to participate in activities and go out with other people; I receive praise and recognition when I do my job well.” Each question is scored from 1 “much less than what I would like” to 5 “as much as I would like.” The maximum total score is 55. Next, the scores are categorized into three groups - low social support (scores 11-26), medium social support (scores 27-41), and high social support (scores 42-55) (Appendix 2).

We also recorded the following items based on data analysis: age was recoded into two groups - “below 60 years” and “60 years and above.” The education level was recoded into “uneducated,” “less than high school,” and “high school or above.” Marital status was recorded as “married” or “unmarried.” Household members were recorded as less than four, four, or more. Income was recorded as “less than 5,000 SR,” “equal to or greater than 5,000 SR.” The number of medications was recorded into two or three medications, four or five medications, and more than five medications. Medication frequency was recorded into one, two, and three or more times. Person who administered your medication was recorded as “me” and “others.”

Prior to the analysis, we conducted data cleaning and assigned codes. The SPSS version 28.0.1.1 (Armonk, NY: IBM Corp.) for windows software was used for data analysis. Descriptive statistics were presented as frequencies and percentages for the categorical variables and as mean ± standard deviation for the numerical variables. Chi-square was used to compare proportions. The level of significance was set to less than 0.05.

## Results

The present study aimed to estimate the prevalence of nonadherent patients having multimorbidity with polypharmacy and its relationship to social support in primary health care centers. Out of the 417 participants, 194 (46.5%) had low medication adherence, 127 (30.5%) had medium medication adherence, and 96 (23%) had high medication adherence. There were 256 (61.4%) male participants, and 161 (38.6%) were females. The mean age was 59.15 (SD ± 11.186) years; 171 (41%) of all participants used two or three medications, 127 (30.5%) used four or five medications, and 119 (28.5%) used more than five medications. Furthermore, 178 (42.7%) participants had two comorbidities, 136 (32.9%) had three comorbidities, 69 (16.5%) had four comorbidities, and 31 (7.5%) had five comorbidities. Table 1 summarizes the characteristic of multiple variables among the participants in relation to adherence.

**Table 1:** Variable characteristics among participants in relation to adherence. SR: Saudi Riyal

Variables		Total, n (%)	High adherence, n (%)	Medium adherence, n (%)	Low adherence, n (%)	p-Value
Medication adherence		417	96 (23%)	127 (30.5%)	194 (46.5%)	-
Age	Below 60 years	187 (100%)	47 (25.1%)	45 (24.1)	95 (50.8%)	0.038
	60 years and above	230 (100%)	49 (21.3%)	82 (35.7%)	99 (43%)	
Gender	Male	256 (100%)	49 (19.1%)	90 (35.2%)	117 (45.7%)	0.010
	Female	161 (100%)	47 (29.2%)	37 (23%)	77 (47.8%)	
Nationality	Saudi	412 (100%)	94 (22.8%)	126 (30.6%)	192 (46.6%)	0.650
	Non-Saudi	5 (100%)	2 (40%)	1 (20%)	2 (40%)	
Smoking status	Smoker	39 (100%)	6 (15.4%)	14 (35.9%)	19 (48.7%)	0.460
	Nonsmoker	378 (100%	90 (23.8%)	113 (29.9%)	175 (46.3%)	
Living situation	With family	407 (100%)	92 (22.6%)	126 (31%)	189 (46.4%)	0.257
	Not with family	10 (100%)	4 (40%)	1 (10%)	5 (50%)	
Marital status	Married	344 (100%)	82 (23.8%)	106 (30.8%)	156 (45.3%)	0.540
	Unmarried	73 (100%)	14 (19.2%)	21 (28.8%)	38 (52.1%)	
Education	Uneducated	101 (100%)	24 (23.8%)	30 (29.7%)	47 (46.5%)	0.883
	Less than high school	169 (100%)	40 (23.7%)	55 (32.5%)	74 (43.8%)	
	High school or above	147 (100%)	32 (21.8%)	42 (28.6%)	73 (49.7%)	
Income	Less than 5000 SR	210 (100%)	43 (20.5%)	65 (31%)	102 (48.6%)	0.448
	5000 SR or more	207 (100%)	53 (25.6%)	62 (30%)	92 (44.4%)	
Household	Less than 4	79 (100%)	17 (21.5%)	19 (24.1%)	43 (54.4%)	0.257
	4 or more	338 (100%)	79 (23.4%)	108 (32%)	151 (44.7%)	
Number of medications	2 or 3	171 (100%)	52 (30.4%)	48 (28.1%)	71 (41.5%)	0.007
	4 or 5	127 (100%)	30 (23.6%)	36 (28.3%)	61 (48%)	
	More than 5	119 (100%)	14 (11.8%)	43 (36.1%)	62 (52.1%)	
Medication frequency	One time	103 (100%)	28 (27.2%)	27 (26.2%)	48 (46.6%)	0.458
	Two times	190 (100%)	46 (24.2%)	58 (30.5%)	86 (45.3%)	
	Three times or more	124 (100%)	22 (17.7%)	42 (33.9%)	60 (48.4%)	
Person who administers your medications	Me	389 (100%)	90 (23.1%)	116 (29.8%)	183 (47%)	0.566
	Others	28 (100%)	6 (21.4%)	11 (39.3%)	11 (39.3%)	
Reasons behind not taking medications	None	253 (100%)	82 (32.4%)	88 (34.8%)	83 (32.8%)	0.000
	Forget	44 (100%)	0 (0%)	8 (18.2%)	36 (81.8%)	
	Fear of adverse events	35 (100%)	6 (17.1%)	15 (42.9%)	14 (40%)	
	No instant relief	24 (100%)	4 (16.7%)	6 (25%)	14 (58.3%)	
	Multiple medications	42 (100%)	4 (9.5%)	7 (16.7%)	31 (73.8%)	
	Do not know when to take it	11 (100%)	0 (0%)	1 (9.1%)	10 (90.9%)	
	Other	8 (100%)	0 (0%)	2 (25%)	6 (75%)	
Number of comorbidities	2	178 (100%)	52 (29.2%)	5(25.3%)	81 (45.5%)	0.087
	3	136 (100%)	27 (19.9%)	42 (30.9%)	67 (49.3%)	
	4	69 (100%)	13 (18.8%)	27 39.1%)	29 (42%)	
	5 or more	31 (100%)	3 (9.7%)	11 (35.5%)	17 (54.8%)	

In terms of medication adherence and its relationship with participant characteristics, participants under 60 years of age displayed higher adherence than those who were 60 years of age and above (25.1% vs. 21.3%, p = 0.038). Women had a higher rate of adherence than men (29.2% vs. 19.1%, p = 0.01). Patients who took two or three medications had higher adherence (30.4%) compared to those who took four or five medications (23.6%) or more than five medications (11.8%, p = 0.007). Among patients with two comorbidities, 29.2% had a high adherence level; among patients with three comorbidities, 19.9% had a high adherence level; among patients with four comorbidities, 18.8% had a high adherence level; and among patients with five comorbidities, 9.7% had a high adherence level (p = 0.087). As for social support (FSSQ), data for 58 (13.9%) participants were missing. There were 246 (59%) participants with high social support, 101 (24.2%) with medium social support, and 12 (2.9%) with low social support. These results are summarized in Table 2.

**Table 2:** Functional Social Support Questionnaire (FSSQ) results.

Total number of respondents	Valid data	High social support	Moderate social support	Low social support	Missing data
417 (100%)	359 (86.1%)	246 (59%)	101 (24.2%)	12 (2.9%)	58 (13.9%)

There were no statistically significant differences between social support and medication adherence. There were 61 (24.8%) participants with high social support and high adherence; 173 (48.2%) participants indicated low social support and low medication adherence (p = 0.470). These results are summarized in Table 3.

**Table 3:** The relation between medication adherence and social support.

	Low adherence	Moderate adherence	High adherence	Total	Total sample	p-Value
Low social support	5 (41.7%)	5 (41.7%)	2 (16.7%)	12 (100%)	359 (100%)	0.470
Moderate social support	53 (52.5%)	31 (30.7%)	17 (16.8%)	101 (100%)		
High social support	115 (46.7%)	70 (28.5%)	62 (24.8%)	246 (100%)		

## Discussion

The objective of the study was to estimate the prevalence of nonadherence to medication in multimorbid patients with polypharmacy, which is 46.5%. We found that the percentage in our study is higher than the percentage in the study conducted by Lozano-Hernández et al., which was 40.8% [15]. The difference in results could be explained by the fact that our study included all adult patients, whereas their study included participants aged between 65 and 74 years. In addition, there was no statistically significant difference between patients’ adherence and social support (p = 0.470) to assess the role of social support in medication adherence (Table 3). On the other hand, most of the participants had high social support (59%) due to the Arabic culture, which involves multiple family gatherings and strong community bonds (Table 2).

Identification of patient characteristics and other factors associated with nonadherence

We found that patients under the age of 60 years have higher adherence than those aged 60 years and above (Table 1). This is primarily because as patients age their cognitive functions deteriorate, which could explain why adherence begins to decline. Furthermore, older adult patients are more likely to seek out nonmedically approved treatments for their illnesses, which may be influenced by relatives or social media. To avoid this problem, further patient education is recommended through social media and television programs that focus on raising patient awareness. The inclusion of a secondary database analysis and pill counts, such as the medication possession ratio (MPR), can also help increase medication adherence [20]. Finally, physicians should be encouraged to establish therapeutic relationships with patients and explain the pathophysiology of the disease, as well as the mechanism of action of the medication to them, so that they can clearly understand their condition and avoid unproven alternative treatments.

Gender is another statistically significant variable associated with medication adherence. In our study, medication adherence was found to be higher in women (29.2%) than in men (19.1%) (Table 1). Another study conducted by Tavares et al. found no relationship between medication adherence and gender [21]. The fact that males comprised the majority of our study’s sample population may have limited the results. Many female patients declined to take part in the study. This could be due to cultural boundaries, so future studies should include female data collectors to avoid this problem.

Most of the participants lived with their families (97.6%), which also contributed to the increased social support in the study. Only 2.3% of the participants lived alone. Additionally, approximately 81% of participants had four or more household members, which contributed to higher social support. Apparently, neither living with a family (p = 0.257) nor having four or more household members (p = 0.257) is statistically significant in terms of medication adherence (Table 1). In Arabic culture, family bonds are strong. When someone’s parents grow old, they care for their parents and live with them. There was no significant relationship between income and medication adherence (p = 0.448). The reason for this could be that all the participants had governmental medical coverage and were given free medication. This finding is consistent with another study by Lozano-Hernández et al. which found a negative relationship between income and medication adherence (p = 0.45) (Table 1) [15].

We noticed that as the number of comorbidities increases the level of adherence decreases, for example, the number of patients with two comorbidities and high adherence is 52 (29.2%), while the number of patients with low adherence is 81 (45.5%). The number of patients with three comorbidities who have high adherence is 27 (19.9%), while the number of patients with low adherence is 67 (49.3%). The level of adherence is inversely proportional to the number of comorbidities, but this relationship is not statistically significant (p = 0.087). For this relationship to be statistically significant, a larger sample may be required. The main reason for this relationship is polypharmacy because the level of adherence is expected to decrease as the number of medications increases. In our study, we found a statistically significant relationship between the number of medications and the level of adherence (p = 0.007). In contrast to our findings, a study conducted in Spain found no relationship between polypharmacy and adherence [15]. On the other hand, a study in Saudi Arabia reported findings similar to ours regarding a relationship that exists between polypharmacy and level of adherence [12]. Family physicians can play an important role in increasing medication adherence among patients having comorbidities with polypharmacy by prescribing the most appropriate medications and avoiding those that are unnecessary. They can also consult with other physicians (for example, cardiologists, rheumatologists, endocrinologists, etc.) to reduce the number of medications, if possible.

Finally, we found that 253 participants claimed they were highly adherent to their medication, while 83 (32.8%) reported low adherence. The most common reason for low adherence, as observed in 44 participants, was that they simply forget to take their medications. As stated earlier, the majority of those with low adherence were over the age of 60 years, hence the adherence results may be, in part, attributed to declining cognitive function among participants in this age group. Multiple medications were the second most common reason for not taking the medication, observed in 42 participants. This issue has been identified in other studies and was supported by our findings, which show that adherence decreases as the number of medications increases. Another reason is the fear of adverse events, which was observed in 35 participants, and 40% of them were low-adherent. Physicians must explain the side effects of each medication, as well as the possibility of them occurring and how to deal with them, to comfort the patient and promote better adherence. Furthermore, a lack of immediate relief is one of the reasons for not taking the medications, which was reported by 24 participants. According to our findings, the most common morbidities were chronic and necessitated long-term management; thus, “no instant relief” can be expected due to the chronicity. Nineteen participants reported not knowing when to take the medications and other nonspecific reasons. Better communication between physicians and their patients can help avoid this problem (Table 1).

Limitations of the study

Although this study produced some useful findings about medication adherence, there were some limitations to the design that must be considered. For example, the results may have been affected by the number of male participants, who made up 61.4% of the patient sample. This imbalance reflects the cultural boundaries that led many potential female patients to refuse participation. Another limitation related to the use of the FSSQ to measure social support. The FSSQ may not be ideal for gauging social support in our culture because most of our community’s families live together. However, living with family does not necessarily imply that patients have strong social support. Hence, a new specific tool to assess social support for our community is necessary for more accurate measurement.

## Conclusions

We found that nonadherence to medication was high in multimorbid patients with polypharmacy (46.5%). However, no statistically significant relationship existed between social support and adherence. In addition, certain patient characteristics were associated with low medication adherence, including age ≥60 years, male gender, and the number of medications per individual. Since the majority of patients did not have a specific reason behind medication nonadherence, further education about the disease complication may increase the level of adherence. Primary healthcare physicians can play an important role in managing nonadherent patients by exploring and managing the different reasons for nonadherence. Finally, short interval appointments with nonadherent patients to assess adherence should be encouraged. Because there is a high level of nonadherence in Riyadh, further attention and action are needed to reach acceptable levels of adherence.

## Appendices

Appendix 1

**Table 4:** Eight-item Morisky Medication Adherence Questionnaire. The table is obtained with permission from Tan et al. [18].

Eight-item Morisky Medication Adherence Questionnaire					
1. Do you sometimes forget to take your medication?	Yes	No	-	-	-
2. Thinking over the past two weeks, were there any days when you did not take your medicine?	Yes	No	-	-	-
3. Have you ever cut back or stopped taking your medication without telling your doctor because you felt worse when you took it?	Yes	No	-	-	-
4. When you travel or leave home, do you sometimes forget to bring along your medication?	Yes	No	-	-	-
5. Did you take all your medicines yesterday?	Yes	No	-	-	-
6. When you feel like your symptoms are under control, do you sometimes stop taking the medicine?	Yes	No	-	-	-
7. Do you ever feel hassled about sticking to your treatment plan?	Yes	No	-	-	-
8. How often do you have difficulty remembering to take all your medications?	Always	Often	Sometimes	Rarely	Never

Appendix 2

**Table 5:** Duke-UNC-11 questionnaire of Functional Social Support. The table is obtained with permission from Broadhead et al. (http://adultmeducation.com/AssessmentTools_4.html) [19].

	Much less than I would like	Less than I would like	Neither much nor little	Almost as much as I would like	As much as I would like
	1	2	3	4	5
1. I have chances to talk to someone I trust about my personal and family					
2. I have chances to talk to someone about money problems					
3. I have chances to talk to someone about problems at work or at home					
4. I receive love and affection					
5. I have people who care what happens to me					
6. I receive help when I am sick in bed					
7. I receive useful advice about important things in my life					
8. I receive help in matters related to my home					
9. I get visits from friends and family					
10. I receive invitations to participate in activities and go out with other people					
11. I receive praise and recognition when I do my job well					
